# In This Issue

**Published:** 1996

## THE CHALLENGE OF DUAL DIAGNOSIS

Alcoholism and psychiatric disorders frequently coexist in the same patient (a condition known as dual diagnosis). Because of the complexity of dual diagnosis, patients with this condition may be misdiagnosed and improperly treated and may “fall through the cracks” of the health care system. Dr. George Woody explores some of the problems involved in diagnosing and treating alcoholics with dual diagnosis. He emphasizes that because each disorder can aggravate the course of the other, both must be treated if the patient is to have the best chance for a good outcome. (pp. 76–80)

## ALCOHOL, ANXIETY, AND DEPRESSIVE DISORDERS

One out of three alcoholics experiences episodes of intense depression and/or severe anxiety. In fact, depressed or anxious alcohol-dependent people often believe that they drink to relieve symptoms of sadness or nervousness. According to Dr. Marc A. Schuckit, however, research does not unanimously support the prior existence of severe depressive or anxiety disorders as a usual cause of alcoholism. Dr. Schuckit reviews recent literature (from family studies, prospective investigations, and studies on children of alcoholics) on the complex interaction between alcohol dependence and long-lasting anxiety or depressive disorders. (pp. 81–85)

## ALCOHOL-USE DISORDER AND SEVERE MENTAL ILLNESS

People with severe mental illnesses, such as schizophrenia, who live in the community (i.e., are not institutionalized) are more than twice as likely to develop an alcohol-use disorder during their lifetime than are people in the general population, report Drs. Robert E. Drake and Kim T. Mueser. Such alcohol abuse frequently exacerbates the psychological and social problems associated with mental disorders and leads to a poorer outcome for patients. The authors review recent findings on the detection, diagnosis, and treatment of patients with dual diagnoses. They contend that integrated treatment combining both alcohol-use disorder and mental health interventions is most effective for these comorbid conditions. (pp. 87–93)

## ALCOHOLISM AND EATING DISORDERS

Women with eating disorders, such as anorexia nervosa and bulimia nervosa, frequently abuse alcohol and other drugs. Alcoholics and bulimics also describe similar feelings of “craving” and a “loss of control” over the substance, become preoccupied with the substance, and repeatedly attempt to stop their pattern of overconsumption. Drs. Lisa R. Lilenfeld and Walter H. Kaye review findings from family studies on the co-occurrence of alcoholism and eating disorders. They describe special considerations for managing alcoholic patients with eating disorders. Although monitoring eating-disordered patients in an alcoholism treatment facility may be challenging and labor intensive, it is vital if treatment for both disorders is to be successful. (pp. 94–99)

## ALCOHOLISM AND ANXIETY DISORDERS: THE ROLE OF FAMILY STUDIES

Many patients with alcoholism also suffer from anxiety disorders—disorders characterized by unrealistic fear, panic, or avoidance behavior. Family studies comparing the prevalence of alcoholism and anxiety disorders among the relatives of people with these disorders are providing useful information on the mechanisms underlying these comorbid conditions. Drs. Kathleen R. Merikangas and Denise Stevens and Ms. Brenda Fenton discuss the results of a Yale University family study indicating that shared genetic or environmental factors may contribute to the development of both alcoholism, especially alcohol dependence, and anxiety disorders. (pp. 100–106)

## DO DRINKING AND SMOKING GO TOGETHER?

It is a common observation that drinkers smoke and smokers drink. In fact, research has confirmed that heavier drinkers do tend to be heavier smokers. Dr. Saul Shiffman and Mr. Mark Balabanis examine the effect of this relationship on alcoholism treatment and smoking cessation. The authors address the concern that attempting or achieving smoking cessation might threaten the success of alcoholism treatment. The authors also explore different theories proposed to explain the alcohol-tobacco link and conclude with recommendations for future research. (pp. 107–110)

## INTERVENTIONS FOR ALCOHOLICS WHO SMOKE

More than 85 percent of adults with a history of alcohol abuse also smoke. In this article, Dr. David Abrams and colleagues discuss the complex interaction that exists between alcohol and tobacco. The authors review methods for helping alcoholics to stop smoking, including taking a proactive role in motivating patients and matching effective interventions to the diverse range of people who are alcoholic smokers. By better understanding the interaction between alcohol and tobacco, scientists can improve treatment outcome and cost-effectiveness for alcoholics who smoke. (pp. 111–117)

## TREATING ALCOHOL PROBLEMS IN THE CONTEXT OF OTHER DRUG ABUSE

People seeking treatment for alcoholism frequently abuse other drugs as well, such as tobacco, cocaine, marijuana, methamphetamine, and opiates. As discussed in this article by Drs. William R. Miller and Melanie E. Bennett, the problem of polydrug use raises important issues for alcoholism treatment providers. A person who abuses multiple drugs may have a more difficult time stopping drinking and a higher risk for relapse to alcohol use after treatment. Conversely, a patient who successfully stops drinking may offset this achievement by substituting another drug in place of alcohol. Successful treatment must take into account both alcohol- and drug-related issues, with particular emphasis on assessment, patient motivation, treatment design, and outcome evaluation. (pp. 118–123)

